# Editorial: Neuronal and Glial Alterations Caused by Viral Infections

**DOI:** 10.3389/fncel.2022.883221

**Published:** 2022-04-05

**Authors:** Bruno Hernáez, Abel Viejo-Borbolla, Jorge Rubén Cabrera

**Affiliations:** ^1^Centro de Biología Molecular Severo Ochoa (Consejo Superior de Investigaciones Científicas and Universidad Autónoma de Madrid), Madrid, Spain; ^2^Institute of Virology, Hannover Medical School, Hanover, Germany; ^3^Excellence Cluster 2155 RESIST, Hannover Medical School, Hanover, Germany; ^4^Labcorp Development, Project Management, Madrid, Spain

**Keywords:** neuroinfection, neuroinflammation, neurodegeneration, virus, glial cells, neuron

Viral infection of peripheral and central nervous systems (PNS and CNS, respectively) is usually accompanied by disease associated with high morbidity and mortality. Neurological disease upon viral infection is the consequence of both direct viral cytopathic effect and the response of the innate and adaptive immune responses. In some instances, even peripheral infection with viruses that do not enter the CNS or PNS results in high expression of cytokines and neuropeptides that cause neuroinflammation, impacting the function of the nervous system, as it has been observed during COVID-19 (Yang et al., 2021).

Certain viruses infect the nervous system as part of their cycle, while others do so only under specific circumstances such as immunosuppression. The consequences of neuroinfection are diverse and tend to be long-lasting. In the most adverse forms viral infection of the nervous system leads to cognitive and motor impairment, and there is a rising discussion whether viruses may participate in neurodegenerative diseases (Eimer et al., 2018; De Chiara et al., 2019; Cabrera et al., 2020; Liu et al., 2021).

Some neurotropic human viruses, like herpes simplex virus (HSV), establish latent infections in peripheral ganglia. However, several reports suggest a role for this virus in cognitive disorders, including Alzheimer's disease (AD), as discussed by Yong et al., in this Special Issue. HSV-1 reactivation in the CNS has also been linked to neurodegeneration (Marcocci et al., 2020) and Prasad et al., explore here the effects of repeated viral antigen stimulation on the activation of microglial cells. On the contrary, most zoonotic viruses replicate lytically in the human nervous system, causing severe disease as discussed by King and Irigoyen for Zika virus (ZIKV). Interestingly, as discussed by Adonis and colleagues in this Special Issue, other zoonotic virus like severe acute respiratory syndrome coronavirus 2 (SARS-CoV-2) can cause posttraumatic stress disorder (PTSD).

The mechanisms that lead to neuroinfection are not well understood for many viruses. Colonization of the PNS and CNS occurs through different routes, including hematogenous transmission and transport within neurons. The role of extracellular vesicles (EV) in viral transmission to the CNS through the blood brain barrier (BBB) and its pathogenic consequences is becoming evident, as discussed in a review by Horn and MacLean. Other viruses, like human alphaherpesviruses, travel inside neurons using cellular motor proteins. In the case of the highly virulent rabies virus (RABV), the most accepted view is that it employs synaptic connections to spread between neurons. Whether this is true or not may require further experimental evidence, as discussed in the review by Beier.

In this “Special Issue”, we assembled five reviews and one original research article that deal with the highly relevant topics mentioned above.

Beier explores the immune evasion mechanisms underlying the RABV infiltration of the CNS and highlights gaps in the most accepted model of RABV spreading, which exclusively considers viral transmission among neurons through synapses. After analysis of evidence supporting or rejecting this synaptically-restricted viral transmission, the initial infection of astrocytes by RABV emerges as an interesting hypothesis to explain the basal interferon-based response detected during infection.

In their mini review, Miranda D. Horn and Andrew G. MacLean summarize the role of EV in viral infection of the CNS, a very interesting, but rather unexplored field. They report on the function of EV during homeostasis and disease, focusing on the misuse of EV by viruses to modulate the immune response, increase virus spread and tropism, facilitating crossing of the BBB, infection of resident CNS cells and modulation of their activity, inducing neuroinflammation and neurodegeneration. More research on this topic is clearly needed to provide novel therapeutic strategies to treat neurological disease caused by viruses.

Some studies have linked re-challenge of neurotropic viruses with neurodegeneration. Prasad et al. (2019) previously identified that antigen specific brain resident memory CD8+ T-cells (bT_RM_) rapidly respond to repeated viral antigen exposure driving to neuroinflammation. They now report that this prolonged antigen re-challenge of bT_RM_ drives to dysregulation of the surrounding microglia, which could have important neurotoxic effects. This study identifies a cellular mechanism that might contribute to neuropathogenesis after reinfection or viral reactivation.

Yong et al. review the current knowledge about the potential involvement of HSV-1 in AD, paying special attention to the neuroanatomical point of view. In this regard, the authors explore the special vulnerability of the hippocampus to HSV-1 infections, as the hippocampus is a key area involved in learning and memory, and one of the main areas affected in AD.

A substantial percentage of survivors of Ebola virus and human immunodeficiency virus infections develop PTSD. This is also observed in about one third of individuals that recover from coronavirus-induced disease 2019 caused by SARS-CoV-2. Sfera et al. thoroughly review the current literature on PTSD, its association with SARS-CoV-2 and discuss the potential mechanisms leading to this neurological disorder in this infection setting. These include the role of BBB leakage, brain derived neurotrophic factor and the fibrinolytic system.

King and Irigoyen debate about the different ZIKV strains and their involvement in the Congenital Zika Syndrome (CZS). Their discussion is focused on the molecular mechanisms underlying CZS and on the potential capacity of ZIKV lineages to employ these mechanisms.

In conclusion, this Special Issue highlights the state-of-the-art and recent advances in viral infection of the nervous system and its pathological consequences. The complexity of this interdisciplinary topic requires the cooperation of virologists, immunologists, and neurologists. Only through this cooperation we will be able to face the challenges ahead in an aging society exposed to old and novel viruses that cause neurological disease.

## Author Contributions

All authors listed have equally contributed to this work and approved it for publication.

## Funding

The laboratory of AV-B was funded by the Deutsche Forschungsgemeinschaft (DFG, German Research Foundation) under Germany's Excellence Strategy – EXC 2155 RESIST – Project ID 39087428. BH lab is funded by the Spanish Ministry of Science and Innovation, and European Union (European Regional Development's Funds, FEDER) grant RTI2018-097581-B-I00.

## Conflict of Interest

JC declares he holds a Project Management position at Labcorp Drug Development. The remaining authors declare that the research was conducted in the absence of any commercial or financial relationships that could be construed as a potential conflict of interest.

## Publisher's Note

All claims expressed in this article are solely those of the authors and do not necessarily represent those of their affiliated organizations, or those of the publisher, the editors and the reviewers. Any product that may be evaluated in this article, or claim that may be made by its manufacturer, is not guaranteed or endorsed by the publisher.
